# *Ethylene-Responsive Transcription Factor 013 *Regulates Physiological and Molecular Responses to Salt Stress in *Arabidopsis thaliana*

**DOI:** 10.3390/antiox15070834

**Published:** 2026-07-01

**Authors:** Rahmatullah Jan, Shahzad Iqbal, Sajad Ali, Muhammad A. Almalki, Mohammad Alfredan, Sajjad Asaf, Kyung-Min Kim

**Affiliations:** 1 Coastal Agriculture Research Institute, Kyungpook National University, Daegu 41566, Republic of Korea; rahmat2021@knu.ac.kr; 2 Department of Semiconductor Engineering, Gachon University, Seongnamdaero, Sujeong-gu, Seongnam-si 13120, Republic of Korea; 3 Department of Biological Sciences, College of Science, King Faisal University, Al-Ahsa 31982, Saudi Arabiamalmalki@kfu.edu.sa (M.A.A.);; 4 Natural and Medical Science Research Center, University of Nizwa, Nizwa 616, Oman; 5 Department of Applied Biosciences, Graduate School, Kyungpook National University, Daegu 41566, Republic of Korea

**Keywords:** abscisic acid, antioxidant defense, *ERF013*, ion homeostasis, salt stress tolerance

## Abstract

Soil salinity severely limits plant growth by disrupting cellular homeostasis and inducing oxidative damage. Although ethylene-responsive transcription factors (ERFs) are central regulators of stress responses, the function of *ERF013* in salt stress responses remains poorly understood. In this study, we investigated the role of *ERF013* in *Arabidopsis thaliana* using *ERF013* overexpression lines (*OE-ERF013*) and genome-edited (*ge-erf013*) under 250 mM NaCl stress, in comparison with wild-type control (CK) and salt-treated wild-type (WT) plants. Under salinity stress, *OE-ERF013* plants maintained vigorous shoot and root growth, exhibiting a 17% increase in shoot fresh weight and a 100% in root fresh weight relative to WT-T plants, whereas *ge-erf013* mutants displayed severe growth inhibition. Salt stress markedly elevated superoxide (O_2_^−^) and hydrogen peroxide (H_2_O_2_) levels in WT-T (62% and 134%) and *ge-erf013* plants (122% and 193%) compared with CK, while *OE-ERF013* plants showed a significant reduction in O_2_^−^·and H_2_O_2_ levels, which decreased by 34% and 64%, respectively, relative to WT-T. Improved redox homeostasis in *OE-ERF013* plants was associated with enhanced catalase (CAT) and superoxide dismutase (SOD) activities (55% and 44%), increased DPPH radical-scavenging activity (62%), maintained total antioxidant capacity (ABTS), and reduced lipid peroxidation, whereas *ge-erf013* plants exhibited a 47% increase in malondialdehyde (MDA) content relative to WT-T. Furthermore, *OE-ERF013* plants displayed reduced electrolyte leakage and sustained higher relative water content (RWC), with only a 15% decline under salt stress. Transcript analysis revealed strong upregulation of key ion homeostasis genes (*SOS1, SOS2, NHX1,* and *HKT1*) in *OE-ERF013* plants, while their expression was suppressed in *ge-erf013* mutants relative to WT-T. Additionally, *OE-ERF013* plants accumulated higher abscisic acid (ABA) levels and showed increased expression of ABA biosynthesis-related genes (*ATAO3* and *ATABA3*), accompanied by enhanced osmotic adjustment through elevated proline, soluble sugars, and sucrose accumulation, as well as improved chlorophyll stability. Collectively, these results demonstrate that *ERF013* acts as a positive regulator of responses to salinity by coordinating ABA signaling, antioxidant defense, ion homeostasis, and osmotic regulation in *Arabidopsis thaliana*.

## 1. Introduction

Soil salinity is one of the most sever abiotic stresses reducing plants’ growth and productivity. More than 20% of irrigated agricultural land is currently affected by salinity, and this proportion is expected to increase due to climate changes and unsustainable agricultural practices [1]. High salinity imposes both osmotic stress and ionic toxicity, leading to growth inhibition, disrupted cellular homeostasis, and premature senescence in plants [2]. To cope with salinity stress, plants have evolved complex and coordinated adaptive mechanisms involving growth modulation, antioxidant defense, ion homeostasis, phytohormone signaling, and metabolic adjustment.

One of the earliest consequences of salt stress is excessive accumulation of reactive oxygen species (ROS), including O_2_^−^ and H_2_O_2_, which cause oxidative damage to lipids, proteins, and nucleic acids [3,4]. To mitigate ROS-induced oxidative stress, plants rely on an efficient antioxidant system consisting of enzymatic components such as SOD, CAT, and POD, as well as non-enzymatic antioxidants and free radical scavengers [5]. In parallel with oxidative stress, salt stress causes ionic imbalance such as excessive Na+ accumulation, which severely disrupts cellular functions. Plant regulate Na+ uptake, transport, and sequestration through well-characterized ion transport systems, including Salt Overly Sensitive (SOS) pathway, vacuolar Na+/H+ antiporters (*NHX*), and high-affinity K+ transporters such as *HKT1* [2,6]. Coordinated regulation of these transporters is essential for maintaining cytosolic ion homeostasis and protecting metabolic processes under saline conditions.

Abscisic acid (ABA) plays a central role in plant responses to salt stress by integrating osmotic stress perception with downstream physiological and molecular responses [2,7]. Salt stress-induced ABA biosynthesis, which, in turn, regulates stress-responsive gene expression, antioxidant defenses, ion transport, and osmotic adjustment [8]. ABA signaling has also been shown to interact with ROS and ion homeostasis pathways, highlighting its role as a key regulatory hub in stress adaptation. In addition to hormonal and ionic regulation, osmotic adjustment through the accumulation of compatible solutes such as proline, soluble sugar, and sucrose is a crucial strategy for maintaining cellular turgor, stabilizing protein and membranes, and scavenging ROS under salt stress [9,10]. Preservation of chlorophyll content and photosynthetic capacity further contributes to sustained growth and biomass accumulation during prolonged exposure to salinity.

Transcription factors play a prominent role in integrating stress-responsive signaling pathways and coordinating adaptive gene expression under adverse environmental conditions. Among them, the APETALA2/ETHYLENE RESPONSIVE FACTOR (Ap2/ERF) family has been widely recognized as a positive regulator of plants’ tolerance to abiotic stresses, including salinity, drought, and oxidative stress [11,12]. ERF proteins function by binding to GCC-box of DRE/CRT cis-elements in the promoters of stress-responsive genes, thereby regulating downstream processes related to redox homeostasis, hormone signaling, ion transport, and metabolic adjustment [13,14,15]. Increasing evidence indicates that the ERF transcription factors enhance salt stress tolerance by promoting ROS detoxification through the activation of antioxidant enzymes such as SOD, CAT, and POD, resulting in reduced ROS accumulation and improved membrane stability [16]. In parallel, ERFs are closely associated with ABA signaling, as several *ERF* genes are induced by ABA and subsequently regulate ABA biosynthetic and ABA-responsive genes, forming positive feedback loops that strengthen stress adaptation and water-use efficiency [8]. Moreover, ERF family members contribute to osmotic adjustment by regulating the accumulation of compatible solutes such as proline and soluble sugars, which help maintain cellular turgor, stabilize membranes, and further mitigate oxidative stress [9].

Numerous members of the *ERF* family have been implicated in the regulation of salinity tolerance in Arabidopsis and other species. For instance, *ERF1* acts as an integrator of ethylene and jasmonate signaling and enhances tolerance to salt and other abiotic stresses through activation of stress-responsive genes [14]. *ERF5* and *ERF6* are rapidly induced by osmotic stress and participate in stress-responsive signaling pathways. *ERF6* regulates the expression of ROS-responsive and stress-related genes, including *STZ*, *WRKY33*, and *MYB51*, suggesting important roles in osmotic and oxidative stress responses [17]. Similarly, *ERF109* has been reported to regulate plants’ responses to salt stress through the modulation of hormone signaling pathways and ROS-related genes, suggesting an important role in stress adaptation [18]. In addition to Arabidopsis, *ERF* transcription factors from rice, wheat, soybean, and cotton have been shown to enhance salt tolerance by regulating ion transport, osmoprotectant accumulation, and the antioxidant defense system [19]. Although numerous AP2/ERF family members have been implicated in salinity tolerance, the function of *ERF013* remains largely unexplored.

In our previous study, we generated and characterized Arabidopsis *ERF013* overexpression and genome-edited lines and demonstrated that *ERF013* plays an important role in plant growth and development [20]. Overexpression of *ERF013* promoted root and shoot growth, enhanced lateral root formation, accelerated flowering, and increased reproductive performance, whereas the genome-edited line exhibited reduced growth and delayed development. These findings suggest that *ERF013* participates in multiple developmental and metabolic pathways. However, despite the well-established roles of several *ERF* family members in abiotic stress responses, the function of *ERF013* in salt stress adaptation remains largely unknown. In particular, it is unclear whether *ERF013* contributes to salinity stress responses through the regulation of antioxidant defense, ABA signaling, osmotic adjustment, and ion homeostasis. Given the central role of *ERF* transcription factors in stress-responsive regulatory networks, we hypothesized that *ERF013* positively regulates salt stress responses by coordinating these physiological and molecular processes. To test this hypothesis, we evaluated *ERF013* overexpression and genome-edited Arabidopsis lines using integrated phenotypic, physiological, biochemical, and molecular analysis under salt stress. Our results demonstrate that *ERF013* enhances responses to salinity by promoting growth and root development; improving antioxidant capacity and redox homeostasis, including ABA accumulation and ABA-responsive gene expression; and activating ion homeostasis-related genes, thereby functioning as a key positive regulator of salt stress adaptation.

## 2. Materials and Methods

### 2.1. Experimental Design

Four experimental groups were established in this study: the wild-type control (CK), the salt-treated wild-type (WT-T), salt-treated *ERF013* overexpression lines (*OE-ERF013*), and salt-treated *ERF013* genome-edited lines (*ge-erf013*). The present study was specifically designed to evaluate the role of *ERF013* in salinity stress; therefore, phenotypic and physiological comparisons were conducted under salt stress conditions to directly assess the stress-responsive functions of *ERF013*. The generation and molecular characterization of the *ERF013* overexpression and genome-edited lines have been reported previously [20]. All plants used were *Arabidopsis thaliana* ecotype Col-0. Plants were grown in soil under controlled environmental conditions (16 h light/8 h dark photoperiod, 25 °C, and 60% relative humidity) [21]. At 20 days after sowing, salt stress was imposed by irrigating plants with a 250 mM salt solution [22]. Prior to stress treatment, all plants were watered with normal tap water. During the stress period, the CK group continued to receive normal water, whereas the remaining treatment groups were supplied with the salt solution. Plants were observed throughout the treatment period, and fully expanded rosette leaves were collected at the same time (in morning around 10.00 am) after two days of salt exposure. Samples were immediately frozen in liquid nitrogen and stored at −80 °C until further analysis. The plant materials were kindly provided by the Plant Molecular Breeding Lab, Kyungpook National University, Republic of Korea.

### 2.2. NBT Staining

For the detection of superoxide radicals (O_2_^−^), fully expanded leaves were collected after two days of salt treatment. Immediately after harvesting, the leaves were submerged in a 0.1%nitro blue tetrazolium (NBT) staining solution and incubated at 25 °C for 16 h in dark conditions. After staining, chlorophyll was removed by incubating the samples in alcohol lactophenol (2:1:1, 95% ethanol: lactic acid: phenol) at 65 °C for 30 min and then rinsed with 50% ethanol until the tissues became transparent. The decolorized leaves were then transferred to a clearing solution consisting of lactic acid, phenol, and water (1:1:1, *v*/*v*/*v*) to improve tissue clarity prior to imaging.

### 2.3. Assessment of H_2_O_2_, O_2_^−^, and Leaf Relative Water Content

The levels of O_2_^−^, H_2_O_2_, and leaf RWC were evaluated two days after the initiation of salt stress. All measurements were conducted using three independent biological replicates. The quantification of O_2_^−^ and H_2_O_2_ was performed according to the procedures described in our previous study [23]. For the determination of RWC, fully expanded leaves were sampled in triplicate following two days of salt treatment. Leaf discs (5 mm in diameter) were excised, and their fresh weight (FW) was recorded immediately. The discs were then floated in 10 mL of distilled water for 3 h at room temperature to achieve full turgidity, after which the turgid weight (TW) was measured. Subsequently, the samples were oven-dried at 70 °C for 24 h to obtain the dry weight (DW).

Leaf relative water content was calculated using the following equation:

$$RWC\;\left(\%\right)=\frac{Fw-DW}{TW-DW}\times 100$$

### 2.4. Determination of Antioxidant Enzyme Activities and Lipid Peroxidation

The activities of CAT, POD, and SOD were determined using fresh leaf tissues harvested two days after the onset of salt stress. Enzyme assays were conducted according to the protocols described in our previous study [24]. All measurements were performed using three biological replicates.

Lipid peroxidation was assessed by quantifying MDA content using a commercial MDA Assay Kit (Sigma, Burlington, MA, USA), following the manufacturer’s guidelines [25]. Briefly, 10 mg of leaf tissue was homogenized on ice in 300 µL of an MDA lysis buffer containing 3 µL of butylated hydroxytoluene (BHT). The homogenate was centrifuged at 13,000 rpm for 10 min, and the resulting supernatant was collected. A 200 µL aliquot of the supernatant was mixed with 600 µL of thiobarbituric acid (TBA) reagent in a 1.5 mL microcentrifuge tube and incubated at 95 °C for 1 h. After incubation, the samples were immediately cooled on ice for 10 min. Subsequently, 200 µL of each reaction mixture, along with the corresponding blank, was transferred to a 96-well microplate, and absorbance was measured at 532 nm using a Shimadzu spectrophotometer (Shimadzu, Kyoto, Japan). Each assay was performed in triplicate.

MDA concentration was calculated using the following equation:

$$C=\frac{S_{a}}{S_{v}}\times \;D$$

where *C* represents the MDA content, *Sₐ* is the amount of MDA detected in the sample, *Sᵥ* is the sample volume, and *D* denotes the dilution factor.

### 2.5. Electrolyte Leakage Measurement

Electrolyte leakage was determined two days after the initiation of salt stress, following a previously reported method with minor modifications [24]. Fresh leaf samples were harvested, and approximately 100 mg of tissue was cut into uniform segments and transferred to test tubes containing 10 mL of deionized distilled water. The tubes were sealed and incubated at 32 °C for 2 h to allow electrolytes to diffuse into the solution.

After incubation, the initial electrical conductivity (EC_1_) of the bathing solution was measured using a conductivity meter (Model CM-115, Kyoto, Japan). To release all remaining electrolytes, the samples were then autoclaved at 120 °C for 20 min. After cooling to room temperature, the final conductivity (EC_2_) was recorded. Electrolyte leakage was expressed as a percentage of total conductivity using the following equation:

$$Electrolyte\;Leakage\;\left(\%\right)=\frac{EC1}{EC2}\times 100$$

### 2.6. Determination of ABTS and DPPH Radical Scavenging Activities

Antioxidant capacity was evaluated using ABTS and DPPH radical scavenging assays two days after salt treatment. All analyses were performed using three biological replicates.

The ABTS assay was conducted based on our previously reported method with minor modifications [23]. Briefly, ABTS radical cations were generated by mixing equal volumes of 7 mM ABTS and 2.4 mM potassium persulfate solutions prepared in double-distilled water. The reaction mixture was incubated in the dark at room temperature for 12 h to allow complete radical formation. The resulting ABTS•^+^ solution was diluted with distilled water (1 mL in 20 mL) to obtain an absorbance of approximately 0.70 at 734 nm.

Leaf extracts were then mixed with 180 µL of the diluted ABTS radical solution, and absorbance was measured at 734 nm using a spectrophotometer (Thermo Fisher Scientific, Vantaa, Finland). ABTS radical scavenging activity was calculated using the following equation:

$$\mathrm{ABTS Scavenging Activity}\left(\%\right)\;=\left(\frac{A_{c}-A_{s}}{A_{c}}\right)\;\times 100\;$$

where *A_(c)_* represents the absorbance of the control and *A_(s)_* represents the absorbance of the sample.

DPPH radical scavenging activity was determined using a 0.1% (*w*/*v*) DPPH solution prepared in methanol. A volume of 100 µL of plant extract was mixed with 100 µL of the DPPH solution in a 96-well microplate and incubated at 25 °C for 30 min in the dark. The control reaction consisted of 100 µL of the DPPH solution mixed with 100 µL of methanol. Absorbance was recorded at 517 nm using the same spectrophotometer. DPPH scavenging activity was calculated according to the following formula:

$$DPPH\;Scavenging\;Activity\;\left(\%\right)\;=[1-\frac{\left(A-A_{0}\right)}{\left(B-B_{0}\right)}]\times 100$$

where *A* is the absorbance of the DPPH–sample mixture, *A*_0_ is the absorbance of methanol containing the sample, *B* is the absorbance of the DPPH control, and *B*_0_ is the absorbance of methanol alone.

### 2.7. RNA Isolation, cDNA Synthesis, and Quantitative Real-Time PCR (qRT-PCR) Analysis

Total RNA was isolated from Arabidopsis tissues using the RNeasy Plant Mini Kit (Qiagen, Valencia, CA, USA) according to the supplier’s protocol. For complementary DNA (cDNA) synthesis, 2 μg of purified RNA was reverse-transcribed using the qPCR-Bio cDNA Synthesis Kit (PCRBIOSYSTEM, Seoul, Republic of Korea). Quantitative real-time PCR (qRT-PCR) assays were carried out with a StepOne™ Plus Real-Time PCR System (Thermo Fisher Scientific, Seoul, Korea) using a SYBR Green-based 2× Real-Time PCR Master Mix (BIOFACT, Daejeon, Korea). The transcript levels of the analyzed genes were quantified relative to the reference gene actin. The primer list and accession numbers of the genes are presented in Supplementary Table S1. Statistical analyses were conducted using ΔCt values obtained from three independent biological replicates. Relative transcript abundance was calculated using the 2^−ΔΔCt^ method and presented as the fold change relative to the control.

### 2.8. Quantification of ABA and Proline

Fresh leaf tissues were harvested two days after salt treatment for the determination of abscisic acid (ABA) content. ABA extraction and quantification were carried out using the Plant Abscisic Acid ELISA Kit (LifeSpan BioSciences, Seattle, WA, USA) in accordance with the manufacturer’s protocol.

Proline levels were quantified in leaf tissues harvested after two days of salt treatment using a modified protocol based on a previously reported method [26,27]. Fresh leaf material (300 mg) was frozen in liquid nitrogen, finely homogenized, and extracted with a 3% sulfosalicylic acid solution. The homogenate was incubated in a boiling water bath for 10 min and subsequently cooled at ambient temperature for approximately 15 min. Following centrifugation at 5000 rpm for 10 min, the obtained supernatant was mixed with 2.5% ninhydrin reagent and glacial acetic acid, then incubated at 100 °C for 1 h. The reaction was stopped by immediately cooling the tubes on ice, after which 4 mL of toluene was added to each sample. The absorbance of the chromophore-containing phase was recorded at 520 nm using a spectrophotometer. Proline concentration was calculated and expressed as µg g^−1^ fresh weight (FW).

### 2.9. Measurement of Soluble Sugar and Sucrose Levels

The contents of total soluble sugars and sucrose were determined from freeze-dried leaf tissues harvested after two days of salt treatment using a previously established procedure [28]. Approximately 0.5 g of lyophilized plant material was pulverized in liquid nitrogen and extracted with 2 mL of 80% (*v*/*v*) ethanol at 80 °C for 20 min. After extraction, samples were centrifuged at 10,000 rpm for 15 min, and the resulting supernatant was carefully recovered while the pellet was discarded. The collected extract was diluted with 4 mL of distilled water and subsequently passed through a 0.2 µm membrane filter prior to analysis. Quantification of soluble sugars and sucrose was carried out using high-performance liquid chromatography (HPLC) on an instrument equipped with a Bio-Rad Aminex 87C column (300 × 7.8 mm). Distilled water served as the mobile phase, which was maintained at a flow rate of 0.6 mL min^−1^ during chromatographic separation.

### 2.10. Chlorophyll Content Analysis

Leaf chlorophyll content was assessed using a SPAD-502 Plus chlorophyll meter (Konica Minolta Sensing, Seoul, Republic of Korea). Readings were recorded from the apical, middle, and basal portions of five leaves for each treatment group. The mean SPAD value obtained from these measurements was subsequently used for statistical analysis.

### 2.11. Statistical Analysis

All experiments were arranged in a completely randomized design and performed with three independent biological replicates. Data were subjected to one-way analysis of variance (ANOVA), and differences among treatment means were assessed using Tukey’s multiple comparison test. Results are expressed as the mean ± standard deviation (SD). Graphical representations were prepared using GraphPad Prism version 10 (GraphPad Software, San Diego, CA, USA).

## 3. Results

### 3.1. ERF013 Regulates Plant Growth and Root Growth Under Salt Stress

**Figure 1:**
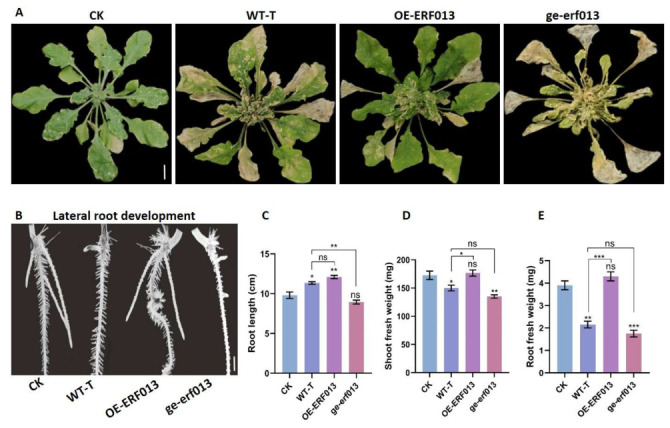
Phenotypic and growth responses of *ERF013* overexpression and genome-edited lines under salt stress. (**A**) Representative rosette phenotypes of CK, WT-T, *OE-ERF013*, and *ge-erf013* lines; image taken after one week of salt exposure. (**B**) Lateral root development of the entire four-treatment group; image taken after one-week growth on salt-included media. (**C**) Shoot fresh weight, (**D**) root fresh weight, and (**E**) root length. Data represent the mean ± SD of three biological replicates. Statistical significance was determined using one-way ANOVA followed by Tukey’s multiple comparison test. Asterisks indicate significant differences (* *p* < 0.05, ** *p* < 0.01, *** *p* < 0.001), while “ns” indicates no significant difference. Scale bar = 1 cm (**A**,**B**).

Salt stress induced clear phenotypic and growth differences among CK, WT-T, *OE-ERF013*, and *ge-erf013* plants (Figure 1). Compared with the CK, the WT-T and *ge-erf013* plants exhibited pronounced salt-induced symptoms, including reduced rosette size, leaf chlorosis, and overall growth inhibition (Figure 1A). In contrast, *OE-ERF013* plants maintained better leaf coloration and overall plant vigor under salt stress. Root phenotypic analysis further revealed distinct responses to salinity (Figure 1B). *OE-ERF013* plants showed enhanced lateral root development, whereas WT-T and *ge-erf013* plants exhibited restricted lateral root growth relative to CK plants. Consistent with these observations, root length increased by 23% in *OE-ERF013* and 15% in WT-T plants but decreased by about 8% in *ge-erf013* plants compared with CK (Figure 1C). When compared with WT-T plants, *OE-ERF013* plants displayed a normal (6%) increase in root length, while *ge-erf013* plants showed a 21% reduction. Quantitative analysis demonstrated that shoot fresh weight was significantly reduced in WT-T and *ge-erf013* plants, whereas *OE-ERF013* plants maintained a shoot fresh weight comparable with that of CK plants (Figure 1D). Relative to WT-T plants, shoot fresh weight increased by approximately 17% in *OE-ERF013* plants but decreased by about 10% in *ge-erf013* plants. A similar trend was observed for root fresh weight (Figure 1E), with significant reductions in WT-T and *ge-erf013* plants compared with CK, while *OE-ERF013* plants showed a non-significant increase relative to CK. Compared with WT-T plants, *OE-ERF013* plants exhibited an approximate 100% increase in root fresh weight. These results demonstrate that *ERF013* plays a positive regulatory role in salt stress responses by promoting root development and maintaining biomass accumulation, whereas loss of ERF013 function increases sensitivity to salinity stress.

### 3.2. ERF013 Regulates Oxidative Stress and Cellular Integrity Under Salt Stress

**Figure 2:**
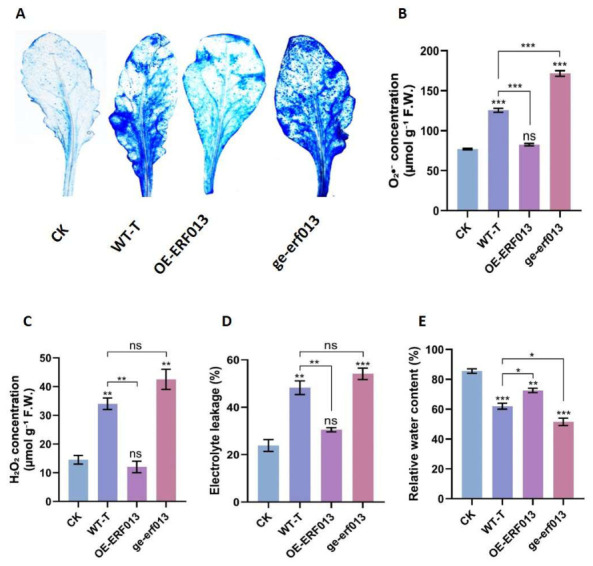
*ERF013* modulates oxidative stress responses and cellular stability under salt stress. (**A**) NBT staining, showing superoxide accumulation in leaves. (**B**) Superoxide (O_2_^−^) concentration, (**C**) hydrogen peroxide (H_2_O_2_) concentration, (**D**) electrolyte leakage percentage, and (**E**) relative water contents. Data represent the mean ± SD of three biological replicates. Statistical significance was determined using one-way ANOVA followed by Tukey’s multiple comparison test. Asterisks indicate significant differences (* *p* < 0.05, ** *p* < 0.01, *** *p* < 0.001), while “ns” indicates no significant difference.

Salt stress markedly affected oxidative stress accumulation and membrane stability in WT-T, *OE-ERF013*, and *ge-erf013* plants (Figure 2). NBT staining revealed weak blue coloration in *OE-ERF013* leaves after two days of salt stress, indicating low superoxide accumulation, whereas WT-T and *ge-erf013* plants exhibited intense staining, suggesting elevated ROS levels (Figure 2A). Consistent with these observations, superoxide (O_2_^−^) content increased by 62% in WT-T and 122% in *ge-erf013* plants relative to CK plants (Figure 2B). In contrast, *OE-ERF013* plants showed only a minor and non-significant increase, maintaining O_2_^−^ levels close to those of CK plants. Moreover, when compared with WT-T plants, *OE-ERF013* plants exhibited a significant reduction in O_2_^−^ content (34%), whereas *ge-erf013* plants showed a significant increase. Similarly, H_2_O_2_ concentration was significantly elevated in WT-T (134%) and *ge-erf013* plants (193%) compared with CK (Figure 2C). In contrast, *OE-ERF013* plants showed no significant increase in H_2_O_2_ accumulation relative to CK, indicating enhanced control of oxidative stress. Compared with WT-T plants, *OE-ERF013* plants exhibited a significant reduction in H_2_O_2_ levels (64%), whereas *ge-erf013* plants showed a comparable, non-significant increase relative to WT-T.

Salt stress also resulted in increased membrane damage, as reflected by electrolyte leakage (Figure 2D). Electrolyte leakage increased by 101% in WT-T and 126% in *ge-erf013* plants compared with CK, whereas *OE-ERF013* plants exhibited only a moderate increase and remained significantly lower than WT-T plants. In agreement with these results, RW declined substantially under salt stress (Figure 2E). WT-T and *ge-erf013* plants showed reductions of 27% and 39%, respectively, relative to CK. By contrast, *OE-ERF013* plants maintained significantly higher RWC, with only a minor reduction of 15% compared with CK. Furthermore, compared with WT-T plants, *OE-ERF013* plants exhibited a 16% increase in RWC, whereas *ge-erf013* plants showed a 16% reduction. Overall, these findings demonstrate that overexpression of *ERF013* mitigates salt-induced oxidative stress, preserves membrane integrity, and enhances water retention, whereas loss of *ERF013* function exacerbates oxidative damage and cellular injury under salt stress.

### 3.3. ERF013 Regulates Antioxidant Defense and Redox Homeostasis Under Salt Stress

**Figure 3:**
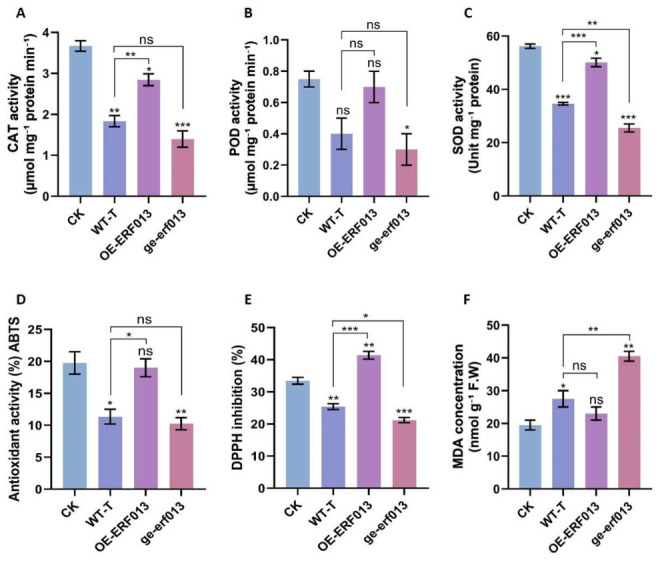
*ERF013* regulates antioxidant enzyme activities, antioxidant capacity, and lipid peroxidation under salt stress. (**A**) Catalase activity (CAT), (**B**) peroxidase activity (POD), (**C**) superoxide dismutase activity (SOD), (**D**) total antioxidant capacity measured by ABTS, (**E**) DPPH radical scavenging activity, and (**F**) malondialdehyde content (MDA). Data represent the mean ± SD of three biological replicates. Statistical significance was determined using one-way ANOVA followed by Tukey’s multiple comparison test. Asterisks indicate significant differences (* *p* < 0.05, ** *p* < 0.01, *** *p* < 0.001), while “ns” indicates no significant difference.

To elucidate the role of *ERF013* in regulating redox homeostasis under salt stress, we analyzed antioxidant enzyme activities, total antioxidant capacity, free radical scavenging activity, and lipid peroxidation levels in WT-T, *OE-ERF013*, and *ge-erf013* plants, compared with CK plants (Figure 3). Salt stress significantly reduced CAT activity in all genotypes; however, the magnitude of the reduction varied markedly among lines (Figure 3A). The strongest decrease was observed in *ge-erf013* plants, followed by WT-T, whereas *OE-ERF013* plants exhibited only a moderate yet significant reduction (22%) relative to CK. Notably, CAT activity in *OE-ERF013* plants was approximately 55% higher than in WT-T plants, while *ge-erf013* plants showed a 23% reduction compared with CK, indicating a compromised H_2_O_2_ detoxification capacity in the mutant background.

POD activity exhibited a comparatively mild response to salt stress (Figure 3B). No significant differences were detected between WT-T or *OE-ERF013* plants and CK, whereas *ge-erf013* plants displayed a pronounced reduction of 60%, suggesting impaired secondary ROS scavenging capacity due to the loss of *ERF013* function. Similarly, SOD activity declined in all genotypes under salt stress relative to CK (Figure 3C). The greatest reduction was observed in *ge-erf013* plants (54%), followed by WT-T (38%), while *OE-ERF013* plants maintained relatively stable SOD activity, showing only a 10% decrease. When compared directly with WT-T under salt stress, SOD activity was 44% higher in *OE-ERF013* plants but 26% lower in *ge-erf013* plants, further supporting a positive regulatory role of *ERF013* in ROS detoxification.

Consistent with enzymatic antioxidant responses, total antioxidant capacity assessed by the ABTS assay declined significantly in WT-T and *ge-erf013* plants under salt stress, whereas *OE-ERF013* plants showed no significant difference compared with CK (Figure 3D). Moreover, ABTS activity in *OE-ERF013* plants was 67% higher than in WT-T plants, indicating enhanced non-enzymatic antioxidant capacity. A similar trend was observed for DPPH radical scavenging activity (Figure 3E). Salt stress significantly reduced DPPH activity in WT-T and *ge-erf013* plants, while *OE-ERF013* plants exhibited a 23% increase relative to CK. Compared with WT-T plants, DPPH activity was 62% higher in *OE-ERF013* plants but 16% lower in *ge-erf013* plants, highlighting the contribution of *ERF013* to maintaining free radical scavenging efficiency. Furthermore, MDA content, an indicator of lipid peroxidation, was significantly induced by salt stress in WT-T and *ge-erf013* plants, whereas *OE-ERF013* plants maintained relatively stable MDA levels compared with CK (Figure 3F). Compared with WT-T, MDA accumulation was moderately lower in *OE-ERF013* plants, while *ge-erf013* plants exhibited a 47% increase, indicating severe oxidative membrane damage in the mutant. All these results demonstrate that *ERF013* positively regulates antioxidant defense systems, enhances both enzymatic and non-enzymatic radical scavenging capacity, and limits oxidative membrane damage under salt stress in Arabidopsis.

### 3.4. ERF013 Positively Regulates the Expression of Ion Homeostasis-Related Genes Under Salt Stress

**Figure 4:**
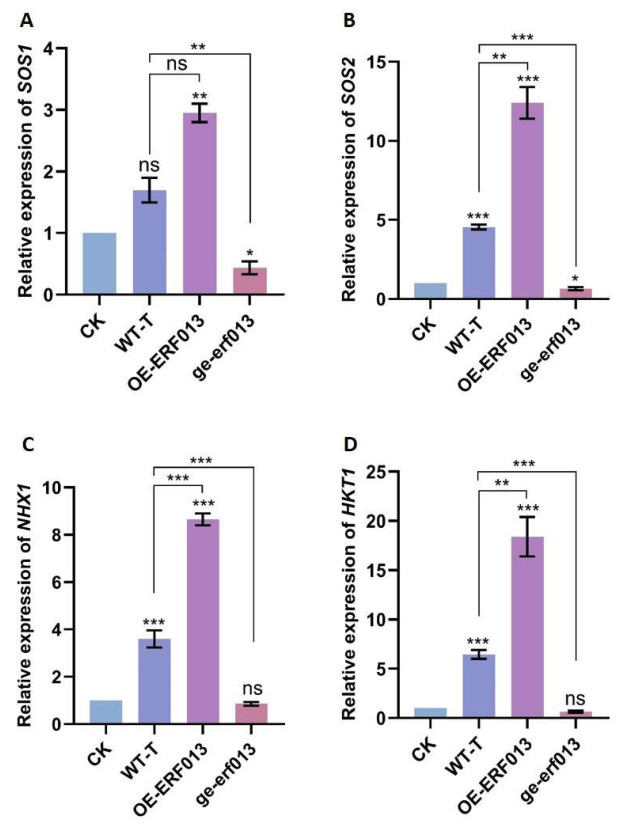
Relative expression of ion homeostasis genes in WT-T, *OE-ERF013*, and *ge-erf013* plants. (**A**) SOS1, (**B**) SOS2, (**C**) NHX1, and (**D**) HKT1 transcript level determined by qRT-PCR. Data represent the mean ± SD of three biological replicates. Statistical significance was determined using one-way ANOVA followed by Tukey’s multiple comparison test. Asterisks indicate significant differences (* *p* < 0.05, ** *p* < 0.01, *** *p* < 0.001), while “ns” indicates no significant difference.

To elucidate the role of *ERF013* in the regulation of ion homeostasis, we analyzed the expression of key Na^+^ transport-related genes in WT-T, *OE-ERF013*, and *ge-erf013* plants under salt stress (Figure 4). The transcript level of *SOS1* was significantly induced in *OE-ERF013* plants (195%), compared with CK, whereas a marked downregulation (56% decrease) was observed in *ge-erf013* plants (Figure 4A). When compared with WT-T plants under salt stress, *OE-ERF013* plants exhibited a further 73% increase (non-significant) in *SOS1* expression, while *ge-erf013* plants showed a 74% significant reduction, indicating a positive regulatory role of *ERF013*. A similar expression pattern was observed for *SOS2* (Figure 4B). Both WT-T and *OE-ERF013* plants displayed significantly higher *SOS2* expression relative to CK, whereas *ge-erf013* plants showed a significant reduction. Notably, under salt stress conditions, *SOS2* expression was increased by 172% in *OE-ERF013* plants but decreased by 85% in *ge-erf013* plants when compared with WT-T plants.

The vacuolar Na^+^/H^+^ antiporter gene *NHX1* and the Na^+^ transporter gene *HKT1* followed a similar trend (Figure 4C,D). The expression of *NHX1* increased by 260% in WT-T plants, while *OE-ERF013* plants exhibited a strong induction of 765% relative to CK (Figure 4C). Likewise, *HKT1* expression was significantly upregulated in WT-T and *OE-ERF013* plants compared with CK, whereas *ge-erf013* plants showed a non-significant reduction (Figure 4D). When compared with WT-T plants, both *NHX1* and *HKT1* were markedly upregulated in *OE-ERF013* plants, while *ge-erf013* plants exhibited significant suppression of these genes. Collectively, these results demonstrate that *ERF013* positively regulates the expression of major ion homeostasis-related genes under salt stress, thereby contributing to enhanced Na^+^ transport and sequestration capacity in plants.

### 3.5. ERF013 Induces ABA and ABA-Responsive Genes Under Salt Stress

**Figure 5:**
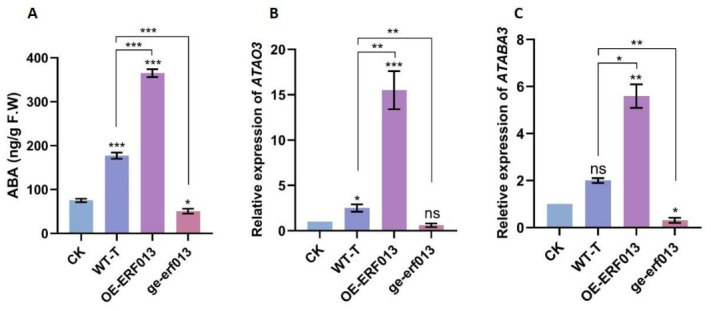
ABA content and expression of ABA-related genes in WT-T, *OE-ERF013*, and *ge-erf013* plants under salt stress. (**A**) Endogenous ABA levels, (**B**) relative expression of *ATAO3*, and (**C**) relative expression of *ATABA3*. Data represent the mean ± SD of three biological replicates. Statistical significance was determined using one-way ANOVA followed by Tukey’s multiple comparison test. Asterisks indicate significant differences (* *p* < 0.05, ** *p* < 0.01, *** *p* < 0.001), while “ns” indicates no significant difference.

To further assess the involvement of *ERF013* in ABA signaling, endogenous ABA accumulation and the expression of ABA-responsive genes were examined in WT-T, *OE-ERF013*, and *ge-erf013* plants under salt stress (Figure 5). Quantification of ABA levels revealed a significant increase in WT-T plants, showing a 136% elevation compared with CK (Figure 5A). Notably, *OE-ERF013* plants accumulated substantially higher ABA, reaching 386% of CK plants’ levels, whereas *ge-erf013* plants exhibited a pronounced reduction, with ABA content decreasing by 32% relative to CK plants. Consistent with these changes in ABA accumulation, the expression of the ABA-responsive gene *ATAO3* was significantly enhanced in *OE-ERF013* plants, while WT-T plants showed only a moderately significant increase compared with CK (Figure 5B). When compared with WT-T plants under salt stress, *ATAO3* expression was strongly induced in *OE-ERF013* plants (520% increase), whereas *ge-erf013* plants exhibited a 76% reduction, although this decrease was not statistically significant.

Similarly, the ABA biosynthesis gene *ATABA3* was markedly upregulated in *OE-ERF013* plants, displaying a 459% increase relative to CK, whereas WT-T plants showed only a moderate and non-significant increase (100%) (Figure 5C). In contrast, *ge-erf013* plants exhibited a significant reduction in *ATABA3* expression compared with CK. Notably, when directly compared with WT-T plants, *ATABA3* expression was significantly higher in *OE-ERF013* plants (179% increase) but strongly suppressed in *ge-erf013* plants (84% reduction), further supporting a positive regulatory role of *ERF013*. Taken together, these findings indicate that *ERF013* promotes ABA accumulation and enhances the expression of ABA-associated genes under salt stress, thereby contributing to the activation of ABA-mediated stress signaling pathways.

### 3.6. ERF013 Regulates Osmolyte Accumulation and Chlorophyll Content Under Salt Stress

**Figure 6:**
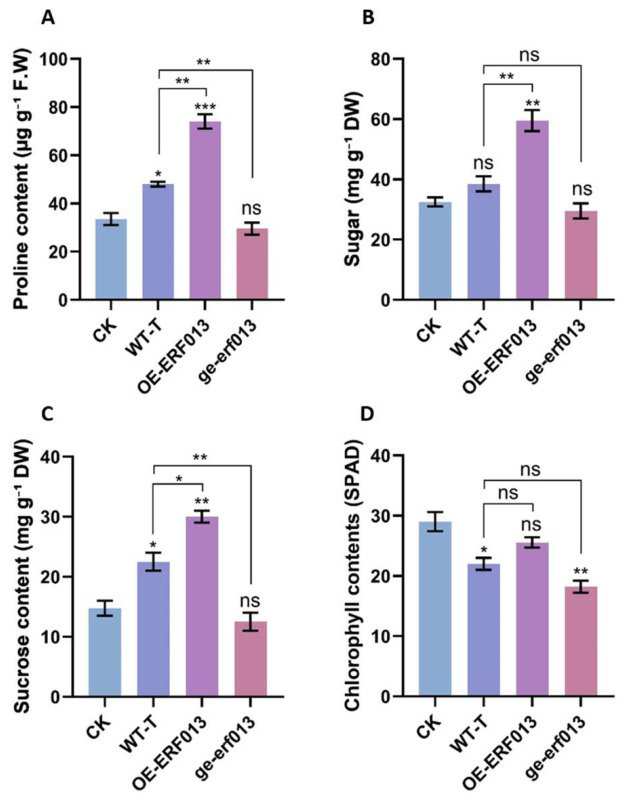
*ERF013* alter osmolyte accumulation and chlorophyll contents in Arabidopsis under salt stress. (**A**) Proline content, (**B**) sugar, (**C**) sucrose, and (**D**) chlorophyll content. Data represent the mean ± SD of three biological replicates. Statistical significance was determined using one-way ANOVA followed by Tukey’s multiple comparison test. Asterisks indicate significant differences (* *p* < 0.05, ** *p* < 0.01, *** *p* < 0.001), while “ns” indicates no significant difference.

Salt stress markedly affected osmolyte accumulation and chlorophyll content in WT-T, *OE-ERF013*, and *ge-erf013* plants compared with control (CK) plants (Figure 6). Under salt stress, proline content increased significantly in WT-T and *OE-ERF013* plants, whereas *ge-erf013* plants exhibited a moderate but non-significant reduction relative to CK (Figure 6A). Notably, *OE-ERF013* plants accumulated 54% more proline than WT-T plants, while *ge-erf013* plants showed a 38% reduction compared with WT-T. These results indicate that *ERF013* overexpression enhances proline accumulation under salt stress, whereas loss of *ERF013* function compromises proline biosynthesis.

Total soluble sugar content also varied significantly among genotypes in response to salt stress (Figure 6B). *OE-ERF013* plants showed a significant increase in sugar accumulation, while WT-T plants exhibited a moderate but non-significant increase, and *ge-erf013* plants displayed a moderate, non-significant reduction compared with CK. Relative to WT-T plants, soluble sugar content increased by 54% in *OE-ERF013* plants but decreased by 23% in *ge-erf013* plants. Similarly, sucrose accumulation was significantly enhanced in WT-T and *OE-ERF013* plants under salt stress compared with CK, whereas *ge-erf013* plants showed a moderate, non-significant reduction (Figure 6C). Compared with WT-T plants, sucrose content increased by 33% in *OE-ERF013* plants, while a pronounced 44% reduction was observed in *ge-erf013* plants under salt stress. Collectively, these findings suggest that ERF013 positively regulates sugar and sucrose accumulation during salt stress, whereas disruption of ERF013 impairs carbohydrate homeostasis.

In contrast, total chlorophyll content declined in all genotypes under salt stress relative to CK (Figure 6D). WT-T and *ge-erf013* plants exhibited significant reductions in chlorophyll content by 24% and 37%, respectively, whereas *OE-ERF013* plants showed only a moderate decrease, indicating improved chlorophyll stability. Overall, these results demonstrate that *ERF013* overexpression enhances osmotic adjustment through increased accumulation of proline and soluble sugars and helps maintain chlorophyll’s integrity under salt stress. Conversely, genome editing of *ERF013* results in impaired osmolyte accumulation and chlorophyll degradation, thereby increasing sensitivity to salt stress.

### 3.7. Correlation Analysis

**Figure 7:**
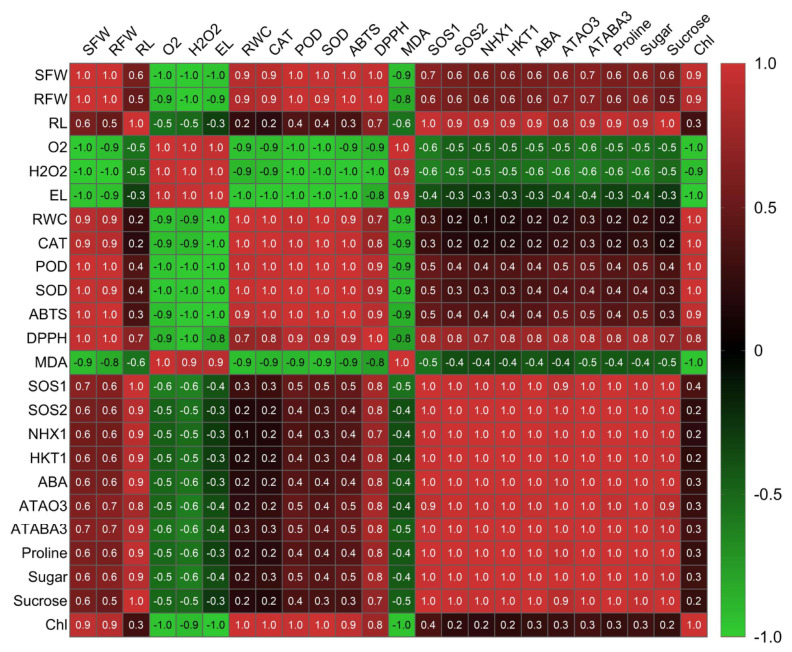
Pearson correlation analysis among physiological traits, antioxidant responses, gene expression, and ABA-associated parameters under salt stress. The heatmap displays Pearson correlation coefficients among growth parameters, oxidative stress indicators, radical scavenging activity, ion homeostasis related-genes, ABA content, ABA-responsive genes, and osmolytes. Red and green colors indicate positive and negative correlations, respectively, with color intensity reflecting correlation strength.

To elucidate the interrelationships among physiological traits, oxidative stress indicators, antioxidant capacity, ion homeostasis, and ABA-related parameters under salt stress, a Pearson correlation analysis was conducted (Figure 7). Growth-associated traits, including shoot fresh weight (SFW), root fresh weight (RFW), relative water content (RWC), and chlorophyll content, were strongly and positively correlated with antioxidant enzyme activities (CAT, POD, and SOD) and osmolyte accumulation (proline, soluble sugars, and sucrose). Conversely, these growth parameters exhibited strong negative correlations with reactive oxygen species (ROS), electrolyte leakage, and lipid peroxidation (MDA), indicating a close association between growth maintenance and oxidative stress mitigation. Genes associated with ion homeostasis (*SOS1*, *SOS2*, *NHX1*, and *HKT1*) showed strong positive correlations with ABA content and ABA-responsive genes (*ATAO3* and *ATABA3*), as well as with antioxidant capacity, while displaying negative correlations with oxidative damage indicators. Notably, ABA accumulation was closely associated with the expression of *NHX1* and *HKT1*, suggesting coordinated regulation between ABA signaling and Na^+^ transport mechanisms under salt stress conditions. Overall, this correlation analysis reveals a tightly integrated regulatory network in which *ERF013*-mediated modulation of antioxidant defenses, ABA signaling, and ion homeostasis collectively underpins enhanced salt stress responses, while effectively limiting oxidative damage.

## 4. Discussion

Plants are constantly challenged by unfavorable environmental conditions, among which soil salinity is one of the most severe abiotic factors restricting plants’ growth, productivity, and geographical distribution worldwide [29]. Salinity stress disturbs cellular homeostasis by imposing osmotic stress, causing ion toxicity, and triggering excessive oxidative damage, which together compromise plants’ growth and survival. To cope with such adverse conditions, plants rely on complex transcriptional regulatory networks that modulate stress-responsive gene expression. Transcription factors play central roles in these networks, enabling plants to perceive stress signals and initiate appropriate adaptive responses. Among them, the AP2/ERF transcription factor family has been widely recognized as a key regulator of plant development and tolerance to abiotic stresses [11,19]. In this study, we provide evidence that *ERF013* functions as a positive regulator of salt stress responses in Arabidopsis. Overexpression of *ERF013* markedly mitigated the inhibitory effects of salinity on plant growth, as reflected by improved overall morphology, enhanced root development, and sustained biomass accumulation under salt stress (Figure 1). Notably, the slight greater root length observed in salt-treated wild-type plants compared with untreated controls may be attributed to the late application of salt stress (20 days after germination), when the root system had already been largely established. Therefore, the measured root length reflects cumulative growth before and after stress exposure and may also represent an adaptive adjustment of root architecture under osmotic stress rather than a growth-promoting effect of salinity. In contrast, genome-edited *erf013* mutants displayed heightened sensitivity to salinity, characterized by reduced rosette size, compromised lateral root formation, and decreased fresh weight. Because the root system architecture plays a crucial role in salt tolerance by determining water uptake efficiency and ion distribution within the plant [1], the enhanced lateral root growth and increased root biomass observed in *OE-ERF013* plants were associated with improved performance under saline conditions.

Salt stress is widely recognized to induce excessive accumulation of ROS, including O_2_^−^ and H_2_O_2_, which can cause severe oxidative damage to cellular membranes, proteins, and nucleic acids [30,31]. In agreement with this, our results demonstrate that *ERF013* plays a pivotal role in maintaining oxidative stress homeostasis under salinity stress (Figure 2). Plants overexpressing *ERF013* accumulated significantly lower levels of O_2_^−^ and H_2_O_2_, exhibited reduced electrolyte leakage, and maintained higher RWC compared with WT-T and genome-edited *erf013* plants. In contrast, disruption of *ERF013* function led to pronounced ROS accumulation, increased membrane damage, and impaired water status, suggesting that *ERF013* contributes to protection against salt-induced oxidative injury. Consistent with our observations, previous studies have reported that ERF transcription factors enhance salt stress responses by promoting ROS detoxification and improving cellular water status. For example, ectopic expression of *LcERF056* in *Arabidopsis* significantly increased relative water content while reducing membrane ion leakage and ROS accumulation under salt stress, indicating that ERF-mediated regulation contributes to improved water retention and reduced oxidative stress [32].

Plants counteract ROS-induced damage through a highly coordinated antioxidant defense system, which includes enzymatic antioxidants such as SOD, CAT, and POD, as well as non-enzymatic antioxidants [33,34,35]. In this study, *ERF013* overexpression markedly enhanced antioxidant capacity under salt stress by sustaining higher activities of SOD, CAT, and POD, together with increased total antioxidant capacity and free radical scavenging activity (Figure 3). By contrast, genome-edited *erf013* plants exhibited a pronounced decline in antioxidant enzyme activities and elevated MDA levels, indicating severe lipid peroxidation. These findings are in line with previous reports showing that overexpression of AP2/ERF genes enhances antioxidant defense under salinity. For instance, overexpression of *OsSTAP1* in rice resulted in increased the activities of SOD, POD, and CAT under salt stress, contributing to improved cellular redox homeostasis and enhanced salt tolerance in transgenic plants compared with wild-type controls [36]. Collectively, these results indicate that *ERF01*3 positively regulates redox homeostasis by strengthening both enzymatic and non-enzymatic antioxidant systems, thereby limiting oxidative membrane damage and enhancing plants’ tolerance to salinity stress. Mechanistically, accumulating evidence indicates that ERF transcription factors can directly regulate ROS-scavenging genes by binding to GCC-box or DRE/CRT cis-elements in the promoters of target genes [37,38]. Notably, *JERF3* was shown to directly bind the GCC-box within the *NtSOD* promoter, thereby activating SOD expression and enhancing salt tolerance in tobacco [39]. Similarly, several ERF family members, including *OsERF71* and *CaERF2*, have been reported to enhance tolerance to abiotic stresses by modulating antioxidant gene expression and ROS detoxification pathways [16,40,41]. In light of these studies and our own findings, it is reasonable to propose that *ERF013* regulates antioxidant defense genes either directly through promoter binding or indirectly via downstream regulatory networks, thereby maintaining ROS homeostasis and protecting plants from salt-induced oxidative stress.

Beyond oxidative stress, ionic imbalance resulting from excessive sodium (Na^+^) accumulation is a major contributor to salt-induced toxicity in plants [1]. To maintain ion homeostasis under saline conditions, plants rely on the Salt Overly Sensitive (SOS) signaling pathway and a set of Na^+^ transporters, including *SOS1*, *SOS2*, *NHX1*, and *HKT1*, which function in Na^+^ extrusion, vacuolar sequestration, and long-distance Na^+^ transport [42,43]. Our transcriptional analyses revealed that *ERF013* positively influences the expression of these key ion homeostasis related genes under salt stress (Figure 4). Specifically, *OE-ERF013* plants exhibited a strong induction of *SOS1*, *SOS2*, *NHX1*, and *HKT1*, whereas the expression of these genes was markedly reduced in genome-edited *erf013* plants. These results suggest that *ERF013* may contribute to ion homeostasis under salt stress through the regulation of genes associated with Na^+^ transport and sequestration.

ABA is a central hormone that mediates plants’ responses to salt and osmotic stress by regulating stomatal closure, osmolyte accumulation, antioxidant defense, and ion homeostasis [7]. In this study, overexpression of *ERF013* resulted in a substantial increase in endogenous ABA levels, accompanied by enhanced expression of ABA biosynthesis and ABA-responsive genes, including *ATABA3* and *ATAO3*, under salt stress conditions (Figure 5). In contrast, genome-edited *erf013* plants exhibited reduced ABA accumulation and compromised expression of ABA-related genes. These results suggests that ERF013 is associated with ABA accumulation and ABA-responsive gene expression under salt stress, consistent with previous studies demonstrating functional crosstalk between ERF transcription factors and ABA pathways during abiotic stress responses [14,15]. Osmotic adjustment represents another critical adaptive mechanism under salt stress, involving the accumulation of compatible solutes such as proline and soluble sugars, which stabilize cellular structures and help maintain turgor pressure [10,44]. Our study demonstrates that *ERF013* overexpression significantly enhanced the accumulation of proline, soluble sugars, and sucrose under salinity stress, whereas disruption of *ERF013* impaired osmolyte accumulation (Figure 6). Moreover, *ERF013-*overexpressing plants maintained higher chlorophyll content under salt stress, suggesting reduced photodamage and improved photosynthetic stability. Collectively, these findings suggest that *ERF013* is involved in hormonal and metabolomic responses associated with enhances salt stress responses.

Taken together, the physiological, biochemical, and molecular data obtained in this study support a model in which *ERF013* acts as a central regulator of salinity responses in Arabidopsis. Overexpression of *ERF013* not only improved plant growth and root development under salt stress but also reduced ROS accumulation, enhanced antioxidant capacity, promoted osmolyte accumulation, increased ABA levels, and induced the expression of genes associated with ion homeostasis and stress adaptation. In contrast, disruption of *ERF013* resulted in impaired stress responses and compromised activation of these protective mechanisms. The coordinated regulation of antioxidant defense, ABA signaling, osmotic adjustment, and Na^+^ homeostasis suggests that *ERF013* functions upstream of multiple stress-responsive pathways rather than resulting in a single protective process. Similar multifunctional roles have been reported for other *ERF* transcriptional factors, which integrate hormonal and environmental signals to optimize plants’ adaptation to adverse conditions [13,15]. Furthermore, the enhanced expression of *ABA3*, *AO3*, *SOS1*, *SOS2*, *NHX1*, and *HKT1* in *ERF013*-overexpressing plants indicates that *ERF013* may coordinate both ABA-dependent and ABA-independent stress signaling networks. Therefore, *ERF013* appears to function as an important transcriptional regulator that enhances salinity responses through the integrated control of redox homeostasis, osmotic protection, hormonal regulation, and ionic balance.

## 5. Conclusions, Limitations, and Future Perspectives

This study demonstrates that *ERF013* functions as a positive regulator of salt stress responses in *Arabidopsis thaliana*. Overexpression of *ERF013* was associated with increased ABA accumulation and enhanced expression of ABA-related genes, together with improved antioxidant capacity, osmotic adjustment, and ion homeostasis under salt stress. These responses were accompanied by reduced oxidative damage and improved growth performance under saline conditions. In contrast, loss of *ERF013* function disrupts redox balance, compromises cellular stability, and increases sensitivity to salt stress. Collectively, these findings identify *ERF013* as an important component of the Arabidopsis salt stress response network. However, although *ERF013* overexpression was associated with increased ABA accumulation, the direct contribution to ABA signaling of *ERF013*-mediated salt responses was not experimentally verified in this study and requires further investigation. Future studies should focus on identifying *ERF013* target genes and regulatory mechanisms using genome-wide approaches and validating its function in crop plants under field-relevant salinity conditions to assess its potential for improving agricultural salt stress responses.

## Data Availability

All data supporting the findings of this study are included within the article.

## Supplementary Materials

The following supporting information can be downloaded at: https://www.mdpi.com/article/10.3390/antiox15070834/s1, Table S1: List of genes, primer sequences, and accession numbers used for gene expression analysis.
