# Supplementary material for: Ethylene-Responsive Transcription Factor 013 Regulates Physiological and Molecular Responses to Salt Stress in Arabidopsis thaliana
Source: Antioxidants (Basel). 2026 Jul 1;15(7):834. doi: 10.3390/antiox15070834 (PMC13404324; doi:10.3390/antiox15070834)
Supplement: Supplementary file 1 [file antioxidants-15-00834-s001.zip › antioxidants-4342490-supplementary.pdf]

Table S1. List of genes, primer sequences, and accession numbers used for gene expression analysis.

Gene Name	Forward primer	Reverse primer	Accession number
SOS1	AGTGTAGGCATTGGTCTGGC	GTTGGCAAGCACAAGCATGA	HE802932.1
SOS2	CACTGCGTTGCAATCCAGAC	CACTTCTGCGGAAAACCACG	HE802949.1
NHX1	CAGTTGTGGTCTTCAACGCG	CCGCTCAAGTCGAAAAGCTG	HE802884.1
HKT1	GCGACACACCGGAGAAACTA	TGGTTCTACCACCCCACTCA	HE802804.1
ATAO3	GGCAACACGGGTACAGGTTA	GCCAAGAAAGCAGCGTTCAA	NM_001336106.1
ATABA3	TTTCCGTGGACCCAAGACAG	TTGCTAAAGGGGGAGCCTTG	NM_101519.3
Actin	CTGCCAGACGGTCAAGTGAT	GAAGCACTTCCTGTGGACGA	U27982.1
